# Characterization of *Listeria monocytogenes* Originating from the Spanish Meat-Processing Chain

**DOI:** 10.3390/foods8110542

**Published:** 2019-11-03

**Authors:** Rosa Capita, Amanda Felices-Mercado, Camino García-Fernández, Carlos Alonso-Calleja

**Affiliations:** 1 Department of Food Hygiene and Technology, Veterinary Faculty, University of León, E-24071 León, Spain; rosa.capita@unileon.es (R.C.); afelim00@estudiantes.unileon.es (A.F.-M.); mc.garcia@unileon.es (C.G.-F.); 2 Institute of Food Science and Technology, University of León, E-24071 León, Spain

**Keywords:** *Listeria monocytogenes*, meat, serotypes, antibiotic resistance, growth kinetic parameters, biofilm

## Abstract

Using agglutination techniques, 118 *Listeria monocytogenes* isolates from red meat and poultry were serotyped. Strains were ascribed to the serotypes 4b/4e (44.1% of the strains), 1/2 (a, b or c; 28.0%), 4c (6.8%), 4d/4e (5.9%) and 3 (a, b or c; 2.5%). Among these are the serotypes most frequently involved in cases of human listeriosis. The susceptibility of 72 strains to 26 antibiotics of clinical importance was determined by disc diffusion (Clinical and Laboratory Standards Institute; CLSI). High levels of resistance were observed to cefoxitin (77.8% of the strains showed resistance), cefotaxime (62.5%), cefepime (73.6%), nalidixic acid (97.2%), nitrofurantoin (51.4%) and oxacillin (93.1%). Less than 3% of the strains showed resistance to the antibiotic classes used in human listeriosis therapy (i.e., ampicillin, gentamicin, rifampicin, chloramphenicol, enrofloxacin, vancomycin, trimethoprim-sulfamethoxazole, erythromycin, and tetracycline). The influence of species and serotype on the growth kinetics (modified Gompertz equation) and on the adhesion ability (crystal violet staining) of nine isolates of *L. monocytogenes* (serotypes 1/2a, 1/2b, 1/2c, 3a, 3b, 3c, 4a, 4b, and 4d), and one strain of *Listeria ivanovii* were investigated. The maximum growth rate (ΔOD_420_-_580_/h) varied between 0.073 ± 0.018 (*L. monocytogenes* 1/2a) and 0.396 ± 0.026 (*L. monocytogenes* 4b). The isolates of *L. monocytogenes* belonging to serotypes 3a and 4a, as well as *L. ivanovii*, showed a greater (*p* < 0.05) biofilm-forming ability than did the remaining strains, including those that belong to the serotypes commonly implied in human listeriosis (1/2a, 1/2b, 1/2c and 4b). The need for training in good hygiene practices during the handling of meat and poultry is highlighted to reduce the risk of human listeriosis.

## 1. Introduction

Twenty species of *Listeria* have been described [1]. Of these, the most prominent is *Listeria monocytogenes*, the species responsible for most cases of human listeriosis, an infection with a high mortality rate, at between 20% and 40% [2]. *Listeriosis* is more frequent and more serious in certain population segments termed at-risk groups, such as “YOPIs”, the Young, the Old, the Pregnant, and the Immunocompromised, where the infection is linked to septicemia, meningitis, or miscarriages [3,4]. Nevertheless, it must be stressed that on occasion, for example after eating food with a high level of contamination, healthy adults have also been affected [5].

The incidence of human listeriosis worldwide ranges between 0.1 and 11.3 cases per million population per year [6]. Most cases of listeriosis are sporadic, and it is estimated that only 5% of cases of human illness are associated with outbreaks [7]. In the USA, approximately 1600 individuals contract listeriosis in its most severe forms each year (the incidence rate is around 0.26 per 100,000 population) and 260 of those affected die because of the infection [8]. Regarding the European Union, 2480 confirmed cases of invasive human listeriosis were reported in 2017 (0.48 cases per 100,000 population). The fatality rate was 13.8% (225 deaths among the 1633 confirmed cases with known outcomes), the highest among all food-borne diseases [9].

*Listeria monocytogenes* is a ubiquitous Gram-positive zoonotic bacterium displaying remarkable resistance to a variety of adverse environmental conditions. This microorganism tolerates high salinity (it can grow in the presence of 10% NaCl) [10] and strong acidity (it can withstand a pH range from 4.1 to 9.6) [11]. Moreover, it should be noted that it can cope with a wide range of temperatures and still grow (from 1 °C to 45 °C, although its optimum growth temperature is between 30 °C and 37 °C), and that it is a facultative anaerobe [10]. These characteristics encourage the appearance and persistence of *L. monocytogenes* in food-processing environments, where the bacterium can remain for long periods of time [11,12].

Control of food-borne pathogenic bacteria requires an awareness of their sources and transmission routes [12]. For this, typing of strains is crucial. Serotyping is based on somatic (O) and flagellar (H) antigens. This technique is normally used to describe a microorganism, as well as in research into the ecological distribution, epidemiology and virulence of strains [13]. Thirteen serotypes have been identified for *L. monocytogenes*, comprising 1/2a, 1/2b, 1/2c, 3a, 3b, 3c, 4a, 4ab, 4b, 4c, 4d, 4e, and 7 [14], but only four of them (1/2a, 1/2b, 1/2c and 4b) are responsible for the majority (approximately 95%) of cases of human illness worldwide [15].

Presently, there is considerable concern for the increased prevalence of antibiotic-resistant bacteria, a problem of critical importance for public health around the world [16]. Although *L. monocytogenes* remains largely sensitive to clinically relevant antimicrobials, a climb in the prevalence of antibiotic-resistant strains has been observed in recent years, particularly in the case of isolates recovered from the food-chain [17,18]. One of the main measures for dealing with the problem of resistance to antibiotics is to have a program for monitoring resistant microorganisms, both in clinical environments and throughout the food-production chain [11,19]. This facilitates identification of trends in the generation of resistance to antibiotics and the planning of strategies for preventing their spread [11].

Biofilms are the predominant mode of microbial growth in nature [20]. They are defined as complex communities of microorganisms, embedded in an extracellular polymer matrix synthesized by the microorganisms themselves, with the ability to adhere to a variety of different biotic or abiotic surfaces [21]. As regards the food industry, once biofilms become established the resident bacteria display enhanced resistance to different environmental stresses, thus encouraging their persistence over long periods of time and increasing the risk of contamination of foodstuffs [22]. Biofilms present on equipment and installation surfaces in the food industry have been identified as the cause of the greater part of outbreaks of food-borne disease [23]. The ability to form biofilms differs from one *L. monocytogenes* strain to another, with major differences having been observed among the various serotypes [24].

The aim of this research work was to gain knowledge of the serotypes and susceptibility to antibiotics of a collection of *L. monocytogenes* isolates from red meat and poultry from North-West Spain, in order to assess the potential dangers posed by these strains for consumers. Additionally, microbial growth parameters and ability to form biofilm was compared for different *Listeria* species and serotypes.

## 2. Materials and Methods

### 2.1. Strains

A total of 118 strains of *L. monocytogenes* were used. These had previously been isolated from red meat (beef and pork) and poultry (chicken and turkey) samples in the Castile and León region (North-West Spain). One strain was selected for each positive sample. Strains were stored at −80 °C in tryptone soya broth (TSB, Oxoid Ltd. Hampshire, UK) with 20% glycerol.

Investigations of growth kinetics and the ability to form biofilm were undertaken with strains from the American Type Culture Collection (ATCC) and the Spanish Type Culture Collection (STCC): *L. monocytogenes* ATCC 19111 (serotype 1/2a), *L. monocytogenes* STCC 936 (serotype 1/2b), *L. monocytogenes* ATCC 19112 (serotype 1/2c), *L. monocytogenes* ATCC 19113 (serotype 3a), *L. monocytogenes* STCC 937 (serotype 3b), *L. monocytogenes* STCC 938 (serotype 3c), *L. monocytogenes* ATCC 19114 (serotype 4a), *L. monocytogenes* ATCC 13932 (serotype 4b), *L. monocytogenes* ATCC 19117 (serotype 4d), and *L. ivanovii* ATCC 19119.

Strains were inoculated into brain heart infusion (BHI, Oxoid) broth and incubated at 37 °C. After 24 h strains were streaked onto tryptone soy agar (TSA, Oxoid) plates, these being incubated at 37 °C for 24 h and then stored at 4 °C while the experiments were performed.

### 2.2. Serotyping

Typing of the 118 strains of *L. monocytogenes* obtained from red meat and poultry was carried out by the agglutination method with the Seiken *Listeria* antisera set (Denka Seiken Co., Tokyo, Japan), in accordance with the manufacturer’s instructions. Twelve antisera were used, eight for determining somatic (O) antigens: I/II, I, IV, V/VI, VI, VII, VIII, and IX, and four for determining flagellar (H) antigens: A, B, C, and D. Strains were grouped into lineages on the basis of their serotype: lineage I, comprising serotypes 1/2b, 3b, 3c, and 4b, lineage II with serotypes 1/2a, 1/2c and 3a, and lineages III and IV, comprising serotypes 4a and 4c [25,26].

### 2.3. Antibiotic Susceptibility Testing

A total of 72 *L. monocytogenes* strains obtained from red meat and poultry were screened for susceptibility to a panel of 26 antimicrobial drugs on Mueller-Hinton agar (Oxoid) by a disc diffusion method. Strains were selected in an attempt to ensure the greatest possible diversity regarding serotypes. The following discs were used: amikacin (AK; 30 µg), streptomycin (STR; 10 µg), gentamycin (CN; 30 µg), kanamycin (K; 30 µg), tobramycin (TOB; 10 µg), rifampicin (RD; 5 µg), imipenem (IPM; 10 µg), cephalothin (KF; 30 µg), cefazolin (KZ; 30 µg), cefoxitin (FOX; 30 µg), cefotaxime (CTX; 30 µg), cefepime (FEP; 30 µg), chloramphenicol (C; 30 µg), enrofloxacin (ENR; 5 µg), ciprofloxacin (CIP; 5 µg), nalidixic acid (NA; 30 µg), vancomycin (VA; 30 µg), amoxicillin-clavulanic acid (AMC; 30 µg), sulbactam-ampicillin (SAM; 20 µg), trimethoprim-sulfamethoxazole (SXT; 25 µg), erythromycin (E; 15 µg), nitrofurantoin (F; 300 µg), ampicillin (AMP; 10 µg), oxacillin (OX; 1 µg), penicillin (P; 10 µg), and tetracycline (TE; 30 µg). All the antibiotic discs were obtained from Oxoid. After incubation at 37 °C for 18–24 h, inhibition zones were measured and scored as sensitive, intermediate (reduced susceptibility) or resistant according to the Clinical and Laboratory Standards Institute (CLSI, Wayne, PA, USA) guidelines [27]. *Staphylococcus aureus* ATCC 29213 and *Escherichia coli* ATCC 25922 were used as reference strains for antibiotic disc control.

### 2.4. Growth Kinetics

For growth curve studies, nine *L. monocytogenes* strains, each of a different serotype, were used, together with one *L. ivanovii* strain. Before use, strains were transferred to TSB and incubated for five hours at 37 °C. These bacterial cultures contained approximately 10^8^ cfu/mL. Three decimal dilutions were performed in TSB. The wells of 100-well polystyrene microtiter plates (Oy Growth Curves Ab Ltd., Helsinki, Finland) were filled with 25 µL of the third dilution of this bacterial culture and 225 µL of TSB to attain a concentration of 10^4^ cfu/mL in the well. Bacterial growth at 37 °C was monitored before incubation (at hour 0) and every hour thereafter until 48 h had elapsed. Growth was determined by measuring the optical density (OD) at 420 nm to 580 nm (OD_420–580_) using a Bioscreen C MBR device (Oy Growth Curves Ab). The micro-well plates were agitated for one minute prior to the measurement of turbidity. The model used to fit growth curves to the data obtained was the modified Gompertz equation [28]: ODt = A + B × exp(-exp(2.71828183 × µ × (L − t)/B + 1)), where t is the time in hours that has elapsed since inoculation, ODt is optical density (determined at 420 nm to 580 nm) at time t, L is the time when the lag period ends (hours), µ is the maximum growth rate achieved (ΔOD_420–580_/h), B is the increase in OD_420–580_ from inoculation to the stationary phase (E), and A is the upper asymptote curve (OD_420–580_ in the stationary stage, E) minus B. The time to stationary phase (T; hours) was calculated as the time required to reach a concentration equal to, or higher than, 99% of the value for E [20]. Values for L, µ, E and T were obtained for each strain and replication by fitting a sigmoidal curve to the data set using a Marquardt algorithm that calculates those parameter values which give the minimum residual sum of squares. Goodness of fit was evaluated using the coefficient of determination (*R*^2^). All experiments were replicated three times on separate days. Each day the experiments were performed in duplicate.

### 2.5. Biofilm Determination

For investigating the production of biofilm, the same strains were used as for the growth curves. Formation of biofilms was determined by measuring optical density at 580 nm (OD_580_) of cells adhering to a microtiter plate (Oy Growth Curves Ab) [20]. Each strain was assessed in triplicate and the procedure was repeated on two separate days. Strains were first incubated in TSB for five hours at 37 °C, until a cell concentration of approximately 10^8^ cfu was attained. Wells were filled with 25 µL of the third dilution of this bacterial culture and 225 µL of TSB to obtain a concentration of 10^4^ cfu/mL. The negative controls (ten in each plate) contained 250 µL of TSB only. The plates were incubated aerobically for 24 h at 37 °C. The contents of the plate were then poured off and the wells washed once with 300 µL of distilled water. The remaining attached bacteria were fixed by adding 250 μL of methanol and letting stand for 15 min at room temperature. The plates were then emptied, air-dried and stained with 250 µL per well of crystal violet (solution at 0.5% in sterile distilled water), letting this stand for five minutes. Excess stain was rinsed off by placing the micro-well plate under running tap water. The plates were air-dried and then the dye bound to the adherent cells was re-solubilized with 250 µL of 33% (*v*/*v*) acetic acid per well, the substance being allowed to work for one minute. Finally, the optical density (OD_580_) was measured in each well using a Bioscreen C MBR (Oy Growth Curves Ab), plates being shaken for one minute before reading. All experiments were replicated three times on separate days. Each day the experiments were performed in duplicate.

The cut-off OD (ODc) was defined as three standard deviations above the mean OD_580_ of the negative controls. Strains were classified into four categories: non-biofilm producers, when OD ≤ ODc, weak biofilm producers, when ODc < OD ≤ (2 × ODc), moderate biofilm producers, when (2 × ODc) < OD ≤ (4 × ODc), or strong biofilm producers, when (4 × ODc) < OD.

### 2.6. Statistical Analysis

The values obtained for each growth parameter (L, µ, E and T) and the OD_580_ (crystal violet assay) were compared for statistical significance using analysis of variance techniques. Mean separations were obtained using Duncan’s multiple range test. Significance was determined at the 5% (*p* < 0.05) level. The Statistica^®^ 8.0 package (Statsoft Ltd., Tulsa, OK, USA) was used for calculations.

## 3. Results and Discussion

### 3.1. Serotyping

**Figure 1:**
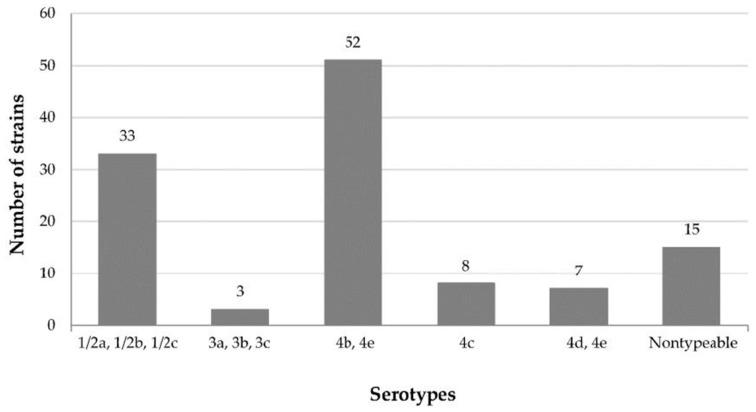
Serotyping for 118 *L. monocytogenes* isolates from red meat and poultry in Spain.

With respect to somatic (O) antigens, agglutination was observed with one or more of the eight antisera in 103 (87.3%) out of the 118 strains studied (Figure 1). No agglutination reactions took place in 15 strains (12.7%), which could not be serotyped. Other researchers also observed strains of *L. monocytogenes* that could not be typed by agglutination techniques [29]. Furthermore, the methods used did not permit distinction of serotype 4e from 4b and 4d, since these strains presented agglutination reactions with the same sera.

Regarding flagellar (H) antigens, no agglutination reactions were observed in any instance. The impossibility of detecting H antigens has also been noted by other authors [30,31,32,33,34]. Antisera for determining flagellar antigens react with the protein flagellin, this being found in the flagella produced by *L. monocytogenes*. The reason there was no agglutination in this work may be that the strains tested presented low or null motility at 25 °C in the culture medium used, BHI with 0.2% agar [33], or that *L. monocytogenes* formed little flagellin [32], or that the weakness of the antigen-antibody reactions makes agglutination barely noticeable [34]. To improve *L. monocytogenes* typing, a multiplex polymerase chain reaction (PCR) assay that separates serotypes into distinct PCR serogroups have been developed [35].

Flagellar (H) antigens permit a distinction between serotypes 1/2a, 1/2b and 1/2c, and also between 3a, 3b, and 3c. Due to the absence of agglutination reactions for the isolates tested here, separation of these strains by serotype was not possible; therefore, these were assigned collectively to Group 1/2 (serotypes 1/2a, 1/2b and 1/2c) or Group 3 (serotypes 3a, 3b, and 3c). Taking this into account, serotyping results are shown in Figure 1, with most strains (82.5% of those typed) being assigned to Groups 1/2 and 4b/4e. These results are worrying, because serotypes 1/2a, 1/2b, 1/2c and 4b are those most often involved in human listeriosis [26]. Strains of Group 1/2 been associated with sporadic cases of listeriosis in Europe and North America, while serotype 4b is responsible for most of the outbreaks of disease [6,36,37].

Strains in Group 1/2, to which 32.0% of the isolates typed were assigned, have often been detected in food, including meat products [12,15,38,39,40,41,42]. Serotype 1/2a is the most prevalent in food-processing environments; thereby indicating its robust ecological adaptability [39]. This serotype appears to contain more plasmids than other serotypes. Since plasmids frequently carry genes that confer resistance to antimicrobial agents, including sanitizers used in processing operations, bacteria harboring such plasmids would have a considerable advantage in these environments [25].

Just 2.9% of strains typed belonged to Group 3. This low percentage agrees with the findings of other researchers, who observed a low prevalence of strains of serotypes 3a, 3b, and 3c both in foodstuffs [15] and among isolates of clinical origin [27,31,38].

Although no strains of serotype 4a were found, several isolates were determined to be of serotypes 4b/4e, 4c and 4d/4e. In fact, the 4b/4e Group accounted for 50.5% of isolates that were successfully typed. This percentage is similar to those noted specifically for *L. monocytogenes* serotype 4b in meat products by Meloni et al. [43] in Italy, Vasilev et al. [44] in Israel, Martins and Germano [45] in Brazil and Fallah et al. [46] in Iran. These authors observed that 50%, 45%, 38% and 45%, respectively, of isolates from foodstuffs belonged to serotype 4b. It should be pointed out that this serotype, besides being the type most often implicated in outbreaks of human listeriosis, appears to have greater potential for virulence than others. Therefore, this serotype is detected more often in patients who have suffered meningitis than in those not having shown any infection in their blood [15]. It should be noted that an important outbreak of listeriosis that occurred in Spain during August and September 2019 by a chilled roasted pork meat product has been linked to *L*. *monocytogenes* serotype 4b [47,48].

Of all strains that could be serotyped here, 7.8% belonged to serotype 4c. Other studies examining *L. monocytogenes* from meat samples have also reported the presence of this serotype [36,38,42,43,44]. It must be pointed out that strains of serotype 4c are associated with animals and are not normally isolated in cases of human listeriosis [36]. The remaining serotype 7 isolates (6.8%) typed here were assigned to the 4d/4e Group. Other researchers have reported the prevalence of these serotypes to be low or absent from foodstuffs [38,40,45,46]. Moreover, these serotypes have not been associated with any cases of human listeriosis [31,39].

### 3.2. Antibiotic Resistance

**Figure 2:**
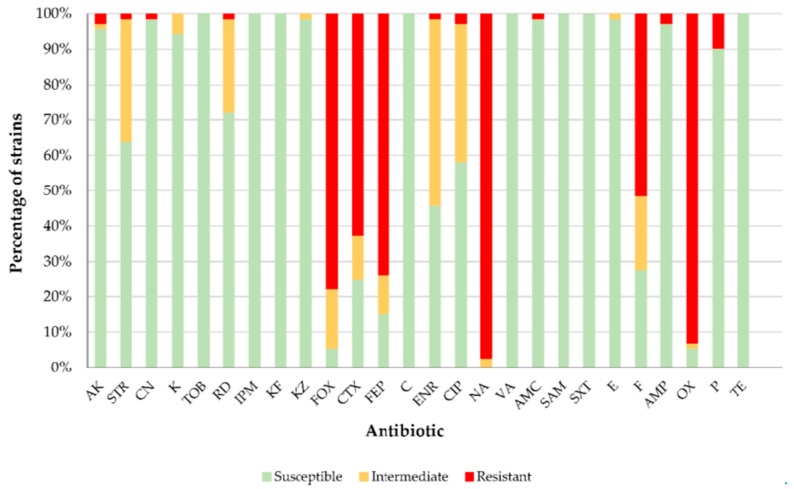
Percentage of *L. monocytogenes* isolates (*n* = 72) from red meat and poultry susceptible (S), intermediate (with reduced susceptibility; I) and resistant (R) to antibiotics. Amikacin (AK), streptomycin (STR), gentamycin (CN), kanamycin (K), tobramycin (TOB), rifampicin (RD), imipenem (IPM), cephalothin (KF), cefazolin (KZ), cefoxitin (FOX), cefotaxime (CTX), cefepime (FEP), chloramphenicol (C), enrofloxacin (ENR), ciprofloxacin (CIP), nalidixic acid (NA), vancomycin (VA), amoxicillin-clavulanic acid (AMC), sulbactam-ampicillin (SAM), trimethoprim-sulfamethoxazole (SXT), erythromycin (E), nitrofurantoin (F), ampicillin (AMP), oxacillin (OX), penicillin (P), tetracycline (TE).

The susceptibility of 72 strains of *L. monocytogenes* obtained from red meat and poultry samples was tested against 26 antibiotics, the results for which are presented in Figure 2. Overall most strains were found to be susceptible to most antibiotics tested. However, for six of the antibiotics there was a high prevalence of resistance among *L. monocytogenes* isolates. These were: cefoxitin (77.8% of strains resistant), cefotaxime (62.5%), cefepime (73.6%), nalidixic acid (97.2%), nitrofurantoin (51.4%) and oxacillin (93.1%). Resistance to one or more of these antibiotics among *Listeria* spp. has been reported by other researchers [17,19,49,50,51,52,53]. While the abovementioned antibiotics are used for treating several infections in humans [54] and animals [55], they are not used in listeriosis therapy, where β-lactams are the antibiotics of first choice, normally ampicillin, administered alone or in combination with gentamicin. In cases of allergy to β-lactams, possible alternatives include erythromycin, vancomycin, trimethoprim/sulfamethoxazole, and fluoroquinolones [17]. Rifampicin, tetracycline and chloramphenicol are also used to treat listeriosis [56]. It should be stressed that several strains showed some resistance to amikacin (2.8% of strains), streptomycin (1.4%), gentamycin (1.4%), rifampicin (1.4%), enrofloxacin (1.4%), ciprofloxacin (2.8%), amoxicillin-clavulanic acid (1.4%), ampicillin (2.8%) and penicillin (9.7%).

In *L. monocytogenes* two MFS (Major Facilitator Superfamily) efflux pumps, MdrL and Lde, have been described to contribute to antibiotic resistance. Lde can confer resistance to hydrophilic fluoroquinolones. Also, within *L. monocytogenes*, MdrL has been described as responsible for benzalkonium resistance when overexpressed [56] and it is able to extrude macrolides, cefotaxime and heavy metals as well [57,58].

Many strains of *L. monocytogenes* show natural resistance to cephalosporins, especially third and fourth generation [19,53]. This fact is borne out by the results of the present research work, in which resistance was noted to cephalosporins, whether second (cefoxitin), third (cefotaxime) or fourth (cefepime) generation. Resistance to nitrofurantoin may be due to the excessive use of this compound in veterinary medicine some years ago [53]. Although today it is a banned substance because of its toxicological risks for consumers, mechanisms for cross-resistance or co-resistance may be related to the presence of resistance to this antimicrobial [16]. The intrinsic resistance of *Listeria* spp. to nalidixic acid was noted some time ago, and in fact this antibiotic is habitually used in selective media for isolating these microorganisms [51,59]. In the present study no strain presented any susceptibility to this antibiotic.

Although *L. monocytogenes* is generally susceptible to a broad range of antibiotics, the emergence of resistant strains observed in recent years is perplexing [46]. Among other causes, this enhancement of resistance may be due to progressive acquisition of antibiotic resistance genes from other bacterial genera through horizontal transmission of mobile genetic elements, such as plasmids or transposons [18].

### 3.3. Growth Kinetics

**Figure 3:**
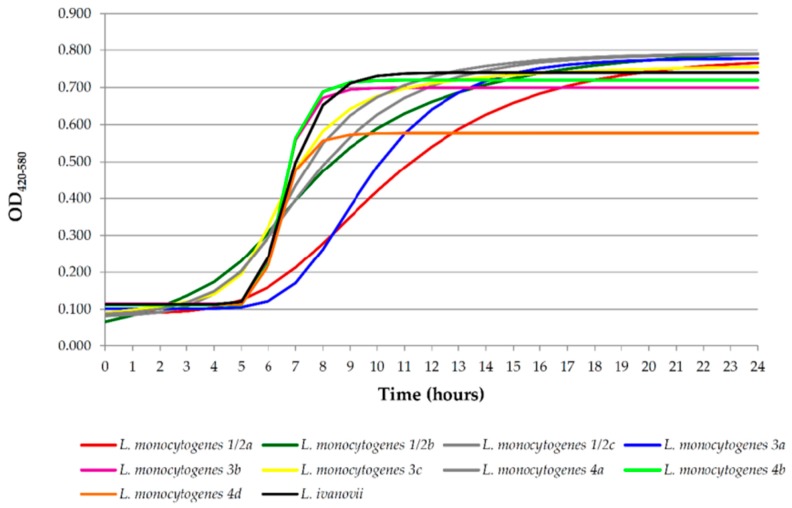
Growth curves (optical density at 420–580 nm) in tryptone soya broth for *Listeria ivanovii* and various serotypes of *Listeria monocytogenes* incubated for 24 h at 37 °C.

Figure 3 shows the growth curves for nine strains of *L. monocytogenes* of various serotypes and one strain of *L. ivanovii*. Table 1 gives the growth parameters for these strains based on the modified Gompertz model. As in other works [60,61,62], the present research revealed great variability among the growth parameters of the different strains of *Listeria* spp. tested. The lag phase (L) reflects the time needed for cells to adapt to a new substrate and begin to multiply [63]. *Listeria monocytogenes* strains of serotypes 3a (at 6.597 ± 0.418 h), 3b (5.717 ± 0.086 h), 4b (5.739 ± 0.111 h) and 4d (5.729 ± 0.133 h) showed a trend to have the longest values for L (*p* < 0.05). In contrast, the *L. monocytogenes* isolate of serotype 1/2b had the shortest duration for L, at 1.839 ± 2.998 h (*p* < 0.05). These findings are similar to those of Vialette et al. [64], who observed that the duration of the lag phases of *L. monocytogenes* of serotypes 1/2b (0.9 ± 0.2 h) and 4b (1.4 ± 0.1 h) were the shortest and longest, respectively, among a set of strains of *L. monocytogenes* as they grew at 20 °C. In another research work, Sant’Ana et al. [65] noted that the average value for L in strains of serotype 4b incubated at 30 °C was 2.4 ± 0.8 h, approximately half that of the strain of that serotype trialed in the current research.

The average L value for the nine strains of *L. monocytogenes* in the current investigation was 4.672 ± 0.971 h. This figure is higher than that observed by Xuan et al. [66], who indicated that the average lag phase at 30 °C was 3.339 ± 0.173 h. It should be noted, however, that the abovementioned authors tested a single strain (ATCC 19114; serotype 4a). The differences in duration of L seen in the various research consulted may be linked to the varying nutrition requirements of the strains [67]. Variations between reports could be also attributed to the different methods applied or the different curve-fitting models used [68].

The strains with greatest growth rates belonged to serotypes 3b (0.387 ± 0.016 ΔOD/h), 4b (0.396 ± 0.026ΔOD/h) and 4d (0.345 ± 0.066 ΔOD/h). Strains of *L. monocytogenes* serotypes 1/2a and 1/2b showed a trend to have the slowest rates of growth (0.073 ± 0.018 and 0.088 ± 0.049 ΔOD/h, respectively). These results coincide with the findings of Pan et al. [24], who observed a higher growth rate in strains of serotype 4b than in those of serotype 1/2a. These are worrying findings taking into account that strains of serotype 4b are frequently associated with outbreaks of human listeriosis. In work by Sant’Ana et al. [65], the average growth rate of strains of serotype 4b incubated at 30 °C was 0.22 ± 0.02 ΔOD/h, lower figures than in the current investigation. In the work being reported here the average growth rate observed for strains of *L. monocytogenes* (0.202 ± 0.042 ΔOD/h) was lower (*p* < 0.05) than for *L. ivanovii* (0.272 ± 0.019 ΔOD/h).

Maximum OD_420–580_ at stationary phase (E) reached by the strains of *Listeria* spp. was an average of 0.738 ± 0.074, lower than noted by other authors consulted. Thus, Mytilinaios et al. [69] observed that maximum OD_600_ was 0.99 when strains were incubated in TSB at 37 °C, and Augustin et al. [60] found an OD_600_ of 0.97 ± 0.06 when strains were incubated at 14.5 °C in tryptone soya yeast extract (TSYE). In the investigation presented here, the highest maximum OD_420–580_ was attained by the strain *L. monocytogenes* serotype 1/2b (0.805 ± 0.031), which also presented the shortest lag phase. This fact suggests that there is an inverse relationship between the length of the lag phase and the maximum growth achieved by the bacterial population. Furthermore, it was also observed that other serotypes with a long lag phase (1/2a, 3a, 3b, 4b, or 4d) attained a relatively limited E (Figure 3). Aguirre and Koutsoumanis [70] had similar findings using a method based on colony forming units counts. These authors observed that the longer the lag phase lasted, the smaller was the population size reached. It must be pointed out that the dimensions of cells may vary between strains and thus cause the differences in OD values observed. Further studies are needed to clarify the reasons for these differences among strains as regards their maximum OD.

The time elapsed before the stationary phase was reached (T) was quite variable between strains, the average values being approximately 14 h. The *L. monocytogenes* 3b, 4b and 4d strains took less time (approximately nine hours) than the remaining strains to reach this phase (*p* < 0.05). The strains presenting the highest values for T were those belonging to serotypes 1/2a (approximately 20 h), 1/2b, 1/2c and 3a (approximately 18 h) and 4a (nearly 16 h). The *L. invanovii* strain took 11 h to reach stationary phase. Initial inoculum levels will influence lag periods and consequently time to stationary phase. Mytilinaios et al. [69] observed that when the inoculum was 5 log units, the time needed to reach the stationary phase when incubated in TSB at 37 °C was 12 h. In contrast, when the starting concentration was 3.3 log_10_ cfu/mL, this value was 16 h. On the basis of the study cited, keeping in mind that the initial concentration of inoculum in the current investigation was approximately 4 log units, it was to be expected that T would be around 14 h, a time corresponding well with the average recorded.

### 3.4. Biofilm Formation

**Figure 4:**
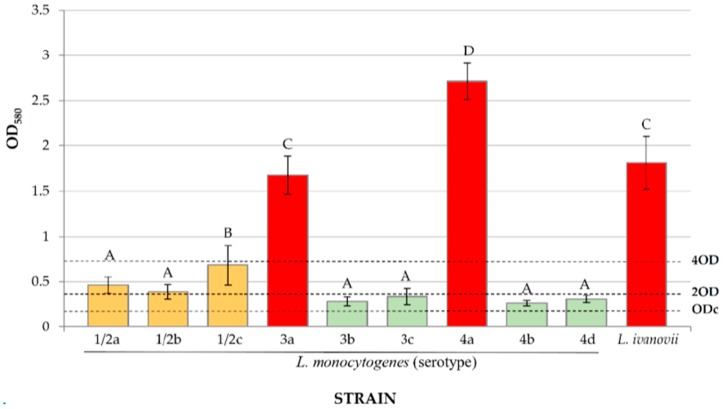
Biofilm formation by various strains of *Listeria* spp. on polystyrene after 24 h of incubation at 37 °C (crystal violet staining technique; optical density at 580 nm). Averages (mean ± standard deviation; *n* = 6) with no letters in common are significantly different (*p* < 0.05). Strong producers of biofilm (OD > 4ODc); moderate producers of biofilm (2ODc < OD ≤ 4ODc); weak producers of biofilm (ODc < OD ≤ 2ODc).

In agreement with other studies [21,71,72] all *Listeria* strains examined here were able to form biofilm under the experimental conditions tested (Figure 4). The mean OD_580_ (crystal violet assay) value for the 10 *Listeria* spp. strains was 0.89 ± 0.13. Even though comparison between reports must be performed with caution because the different methodologies used, our results were similar to the OD_580_ measurements of 0.8 reported by Kadam et al. [73], who examined biofilm formation for 143 *L. monocytogenes* strains after incubation at 37 °C for 24 h in TSB. The average OD_580_ found in the present study fit in the wide range of values observed by Nilsson et al. [74] for a collection of 95 *L. monocytogenes* strains incubated at 37 °C for 24 h, where OD_595_ values ranged from 0.02 to 1.68. These authors used BHI, which is more nutritious than TSB. It should be noted that the ability to form biofilm may vary as a function of availability of nutrients [74].

*L. monocytogenes* strains of serotypes 3a and 4a, and *L. ivanovii* were strong producers of biofilm, with significant differences (*p* < 0.05) relative to the remaining strains. The OD_580_ observed for *L. ivanovii* in the present investigation was 1.81 ± 0.29, a value similar to that obtained by Nyenje et al. [75], where OD_595_ was 1.754 ± 0.763. There is little information available regarding the biofilm-forming capacity of *L. ivanovii*. This is most likely because this bacterium appears to have low virulence potential in humans as demonstrated by the few cases of human listeriosis reported. It should be noted that in the present study the OD_580_ for *L. monocytogenes* (0.79 ± 0.82; average figure for all the strains trialed) was lower (*p* < 0.01) than the OD_580_ for *L. ivanovii* (1.81 ± 0.29).

*Listeria monocytogenes* strains of the serotypes 1/2a, 1/2b and 1/2c gave rise to the formation of moderate amounts of biofilm, while the strain of serotype 4b was a weak biofilm producer; albeit it should be noted that the observed differences between values were not significant (*p* > 0.05). The greater production of biofilm by isolates of Group 1/2 relative to serotype 4b is a finding in agreement with the results of other authors [24,73,74]. Strains 3b, 3c, 4b, and 4d showed only weak production of biofilm. The fact that the serotype most frequently involved in outbreaks of human listeriosis (4b) has a weak ability to form biofilms is a favorable finding in the food safety scenario. 

Based on subtyping techniques, *L. monocytogenes* can be classified into large genetic groups with differing characteristics, termed lineages [76]. Most cases of human listeriosis are associated with lineages I and II [25,76,77,78]. Specifically, cases forming part of outbreaks are related to lineage I, while sporadic cases are linked to lineage II [77]. It should be pointed out that strains of serotypes 3b, 3c, and 4b, which showed the least capacity for biofilm production, belong to lineage I, which is characterized by having very slight genetic diversity [25,76,78]. Lineage II is made up of serotypes 1/2a, 1/2c and 3a, and is characterized by its great genetic diversity [25,66]. As may be seen in Figure 4, these last three serotypes formed more biofilm than those in lineage I. Serotype 4a, which produced the greatest amount of biofilm in this study, belongs to lineage III. Serotype 4a has been isolated principally from ruminants and other non-primate mammals, and has not been linked to cases of human listeriosis [25,77].

It is likely that the ability to form biofilms is linked to virulence genes, since in this investigation it was found that the more virulent a strain was the less able to form biofilm and vice versa. This same observation has been made by other authors [23,79,80], who found increased formation of biofilm in strains of *L. monocytogenes* that had less virulence. The same hypothesis would explain why in this study the strain of *L. ivanovii* formed significantly more (*p* < 0.01) biofilm than the average for *L. monocytogenes*. It should be noted, however, that no virulence testing was performed in this research work. Moreover, the limited number of strains tested prevents any strong conclusions.

## 4. Conclusions

Of the *L. monocytogenes* strains from red meat and poultry that could be serotyped (87.3%), the highest prevalence corresponded to serotypes 4b/4e. This is troubling since serotype 4b is the most frequently involved in outbreaks of human listeriosis. High levels of resistance to cephalosporins (cefoxitin, cefotaxime, cefepime), nalidixic acid, nitrofurantoin, and oxacillin were observed. It is true that *L. monocytogenes* presents intrinsic resistance to several of these antibiotics. However, in view of the seriousness of human listeriosis, the findings in this research make it advisable to set in place measures for monitoring and control that will permit any increase in the resistance to antibiotics of this bacterium to be avoided. Striking differences were noted in the growth parameters of the strains of *Listeria* as a function of species and serotype. The highest lag phase values were observed for serotypes 3a, 3b, 4b, and 4d of *L. monocytogenes*, while the strain of serotype 1/2b presented the shortest values for this kinetic parameter. The growth rate of the *L. ivanovii* strain tested here was greater than that of *L. monocytogenes* (average values). The results obtained highlighted an inverse relationship between the duration of the lag phase and the maximum density of bacteria in the stationary phase. The average time elapsed to stationary phase was 14 h. The strains of *L. monocytogenes* studied were strong (22.2%), moderate (33.3%) or weak (44.4%) biofilm producers. *Listeria ivanovii* showed a much greater capacity to form biofilm than did *L. monocytogenes*, a result that suggests an inverse relationship between virulence and ability to produce biofilm.

## Figures and Tables

**Table 1:** Microbial growth parameters recorded for ten strains of *Listeria* spp. incubated in tryptone soy broth for 24 h at 37 °C.

Strain	Growth Kinetic Parameters			
	L	µ	E	T
*Listeria monocytogenes* 1/2a	5.319 ± 0.504 ^cde^	0.073 ± 0.018 ^a^	0.781 ± 0.015 ^de^	19.800 ± 0.447 ^d^
*Listeria monocytogenes* 1/2b	1.839 ± 2.998 ^a^	0.088 ± 0.049 ^ab^	0.805 ± 0.031 ^e^	18.000 ± 2.646 ^cd^
*Listeria monocytogenes* 1/2c	3.765 ± 0.957 ^bc^	0.103 ± 0.030 ^abc^	0.790 ± 0.038 ^e^	17.600 ± 2.074 ^cd^
*Listeria monocytogenes* 3a	6.597 ± 0.418 ^e^	0.118 ± 0.022 ^abc^	0.778 ± 0.024 ^de^	17.800 ± 1.304 ^cd^
*Listeria monocytogenes* 3b	5.717 ± 0.086 ^e^	0.387 ± 0.016 ^e^	0.699 ± 0.053 ^b^	9.167 ± 0.408 ^a^
*Listeria monocytogenes* 3c	3.479 ± 2.846 ^b^	0.164 ± 0.078 ^c^	0.761 ± 0.024 ^cde^	14.000 ± 3.674 ^b^
*Listeria monocytogenes* 4a	3.864 ± 1.656 ^bcd^	0.144 ± 0.081 ^bc^	0.793 ± 0.037 ^e^	15.600 ± 3.435 ^bc^
*Listeria monocytogenes* 4b	5.739 ± 0.111 ^e^	0.396 ± 0.026 ^e^	0.719 ± 0.033 ^bc^	9.167 ± 0.408 ^a^
*Listeria monocytogenes* 4d	5.729 ± 0.133 ^e^	0.345 ± 0.066 ^e^	0.578 ± 0.049 ^a^	9.000 ± 0.894 ^a^
*Listeria ivanovii*	5.528 ± 0.226 ^de^	0.272 ± 0.019 ^d^	0.740 ± 0.023 ^bcd^	11.000 ± 0.000 ^a^

L, lag phase (h); µ, maximum growth rate (ΔDO/h); E, maximum optical density (OD; determined at 420–580 nm); T, time (h) elapsed to stationary phase. Each value is the mean of six determinations. Average values in the same column without any letter in common are significantly different (*p* < 0.05).
